# The effects of aging and maternal protein restriction during lactation on thymic involution and peripheral immunosenescence in adult mice

**DOI:** 10.18632/oncotarget.7176

**Published:** 2016-02-03

**Authors:** Chantal A. A. Heppolette, Jian-Hua Chen, Sarah K. Carr, Donald B. Palmer, Susan E. Ozanne

**Affiliations:** ^1^ University of Cambridge Metabolic Research Laboratories and MRC Metabolic Diseases Unit, Wellcome Trust-MRC Institute of Metabolic Science, Addenbrooke's Hospital, Cambridge, UK; ^2^ Department of Comparative Biomedical Sciences, Royal Veterinary College, University of London, London, UK

**Keywords:** developmental programming, immunosenescence, lifespan, maternal diet, thymic involution, Gerotarget

## Abstract

Environmental factors such as nutrition during early life can influence long-term health, a concept termed developmental programming. Initial research was focused towards the effects on metabolic health but more recent studies have demonstrated effects on parameters such as lifespan and immunity. In this study we report that maternal protein restriction during lactation in mice, that is known to prolong lifespan, slows aging of the central and peripheral immune systems. Offspring of dams fed a postnatal low-protein (PLP) diet during lactation had a significant increase in thymic cellularity and T cell numbers across their lifespan compared to controls, and a less marked age-associated decrease in thymocyte cluster of differentiation (CD) 3 expression. PLP animals also demonstrated increased relative splenic cellularity, increased naïve: memory CD4^+^ and CD8^+^ T cell ratios, increased staining and density of germinal centres, and decreased gene expression of p16 in the spleen, a robust biomarker of aging. A slower rate of splenic aging in PLP animals would be expected to result in decreased susceptibility to infection and neoplasia. In conclusion nutritionally-induced slow postnatal growth leads to delayed aging of the adaptive immune system, which may contribute towards the extended lifespan observed in these animals.

## INTRODUCTION

The developmental origins of health and disease hypothesis describes the phenomenon whereby exposures *in utero* and early postnatal life modulate long-term health [1]. Original studies focused on how early life events increase the susceptibility to metabolic diseases such as type 2 diabetes and cardiovascular disease [2-4]. However accumulating evidence suggests a role for maternal exposures on non-metabolic disease parameters such as psychiatric disorders, osteoporosis and immune health [5-7]. Interventions can also be beneficial, evidenced by numerous studies showing that the intake of breast milk significantly reduces the risk of developing metabolic syndrome in later life [8].

The development of the adaptive immune system in mammals occurs during fetal and early postnatal growth periods, during which they are vulnerable to environmental insults [9]. Epidemiological data from rural Gambia have shown that infants born during the hungry season where food supplies are limited, have a smaller thymic volume and are more prone to infection compared to those born in the harvest season [6, 10]. Another study in humans observed that a longer period of breastfeeding increased thymic volume in infants, which is indicative of increased immune capacity [11]. Studies using animal models also support the concept that early nutrition has an effect on the immune system. For example, Osgerby and co-workers observed reduced thymic mass in fetal sheep at various stages of gestation in underfed mothers [12]. Previous work in our laboratory revealed that offspring born to normally fed dams and suckled by protein restricted dams (postnatal low protein: PLP offspring), demonstrated slow growth during lactation, extended longevity and continued growth of the thymus between 21 days and 3 months (which was not apparent in control animals) [13, 14].

An efficient and functional adaptive immune system is considered vital for protection against infection, autoimmune disease and tumour growth, and comprises of T and B lymphocytes, which originate in the thymus and bone marrow respectively. In thymocyte development, stem cells derived from bone marrow enter the thymus via the cortico-medullary junction (CMJ) where they commit to the T cell fate. These thymocytes which are double negative (DN) for both co-receptors CD4 and CD8, migrate to the cortex where they progress through four developmental stages characterised by the expression of CD25 and CD44: DN1 (CD44^+^ CD25^−^), DN2 (CD44^+^ CD25^+^), DN3 (CD44^−^ CD25^+^) and DN4 (CD44^−^ CD25^−^) [15]. The magnitude of T cell receptor (TCR identified by CD3 marker) expression increases on cells throughout thymocyte development [16]. Co-receptor expression is then upregulated forming double positive (DP) CD4^+^ CD8^+^ thymocytes [17] and subsequently these cells differentiate into either single positive (SP) CD4^+^ or SP CD8^+^ thymocytes, which migrate to the medulla and are released into the periphery [18]. Newly developed T and B lymphocytes migrate to secondary lymph nodes such as the spleen, whereby following activation, they initiate an effector immune response.

The immune system becomes impaired with increasing age, which is termed immunosenescence, and accounts for the increase in immune related diseases observed in the elderly [19]. One of the most notable changes is the involution of the thymus which occurs with increasing age, and is characterized by a reduction in size, epithelial space and cellularity. Together with a disorganized microenvironment, this results in a reduction in T cell output [20]. Such alterations in the thymus lead to a reduction in number and diversity of naïve T cells in the periphery, leading to an age-associated increase in memory T cells [21]. These characteristics directly contribute towards the increased susceptibility to infection and neoplasia in the elderly population. Therefore environmental exposures during early life that can delay the development of immunosenescence are likely to have large implications for the healthspan as well as the lifespan of an individual.

In the current study we tested the hypothesis that immunosenescence can be influenced by early life events. We proposed that animals exposed to a maternal low protein diet during lactation, which are known to have an increased lifespan, would demonstrate delayed aging of the immune system compared to control offspring. To address this hypothesis we studied several immune parameters across the lifespan in PLP and control mice. The effect of a PLP diet was analyzed in both the thymus and spleen, two key immune tissues.

## RESULTS

### Body weight

**Figure 1:**
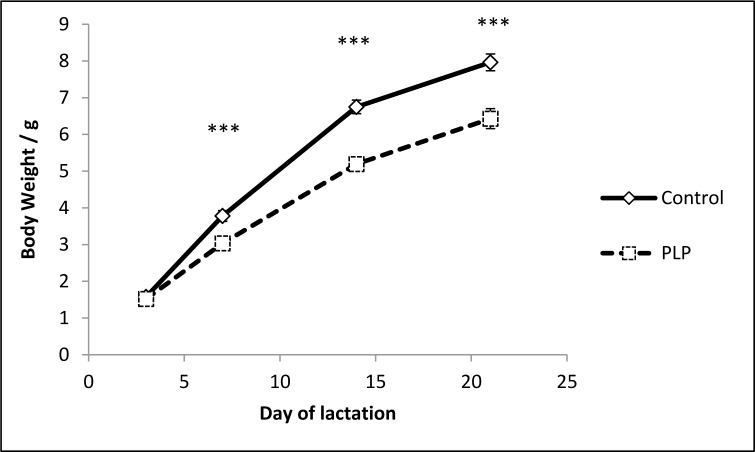
Effect of maternal diet on body weight during lactation Body weights of offspring were measured at day 3, 7, 14 and 21 and were consistently lower from day 7 onwards in the PLP group (***p < 0.001) compared to control animals. *n* = 16-30 per group. Data are represented as mean +/− SEM.

The body weight of pups was recorded from birth and throughout their lifespan. Pups suckled by dams fed a PLP diet grew slowly during the lactation period and were significantly smaller compared to control offspring from day 7 onwards (*p* < 0.001) (Figure 1) as described previously [22]. This difference in body weight was maintained throughout their lifespan (Table 1).

**Table 1:** Effect of maternal diet on body weight during lifespan

Age	Diet	Mean body weight / g
21d	Control	7.96 ± 0.23
	PLP	6.43 ± 0.27 ***
3m	Control	28.14 ± 0.50
	PLP	26.52 ± 0.40 **
18m	Control	44.90 ± 1.30
	PLP	41.66 ± 2.30 *
23m	Control	41.11 ± 1.90
	PLP	33.70 ± 5.70 **

Body weights of offspring were measured weekly during their lifespan and were consistently lower in the PLP group compared to control animals at 21d (***p<0.001), 3m (**p<0.01), 18m (*p<0.05) and 23m (**p<0.01). n = 16-30 per group. Data are represented as mean +/− SEM.

### Thymic cellularity

**Figure 2:**
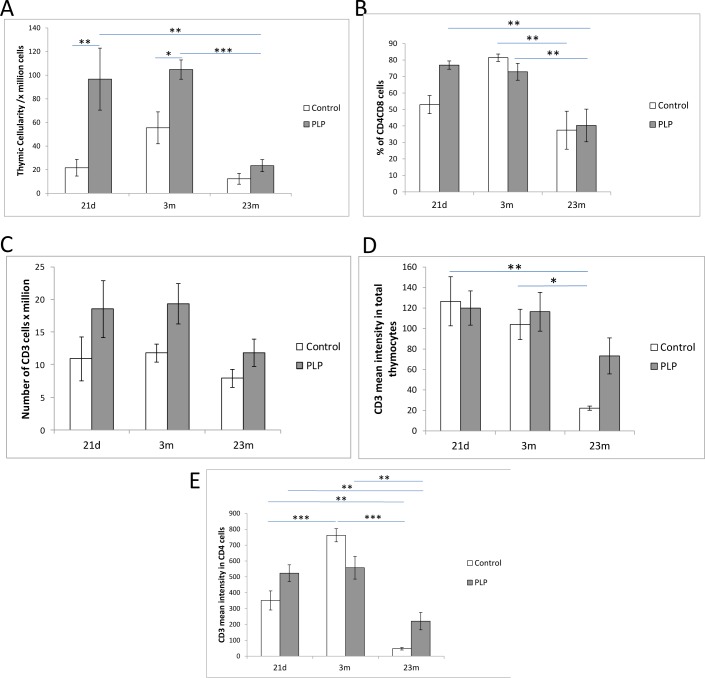
Effect of age and maternal diet on thymic cellularity and development **A.** Thymic cellularity was significantly affected by age (*p* < 0.001). PLP offspring had significantly higher thymic cellularity compared to control animals (*p* < 0.001) especially at 21d and 3m. **B.** The proportion of DP CD4^+^ CD8^+^ cells as measured by flow cytometry, significantly decreased with age (*p* < 0.001). However there was no effect of maternal diet. **C.** The number of CD3^+^ thymocytes were significantly higher in PLP offspring compared to control animals (*p* < 0.05). However there was no significant effect of age on these cells. **D.** The density of CD3 expression on total thymocytes decreased significantly with age (*p* < 0.01). **E.** The level of CD3 expression in CD4^+^ cells peaked at 3m, and decreased by 23m of age (*p* < 0.001). However this change with age was less substantial in PLP animals (*p* < 0.05). *p < 0.05, **p < 0.01, ***p < 0.001. *n* = 5-9 per group. Data are represented as mean +/− SEM.

Concurrent to previous findings, thymic cellularity was significantly affected by age (*p* < 0.001) (Figure 2A) [20]. Given that our previous study demonstrated an effect of maternal diet on thymic weight, we further investigated the effect of maternal diet on thymic cellularity [13]. Here we observed that thymic cellularity was significantly higher in PLP animals compared to the control group (effect of maternal diet *p* < 0.001) especially at 21d and 3m of age (Figure 2A).

### Thymocyte subsets

Given the changes observed in thymic cellularity, the T cell population was analysed in more detail. Using CD4 and CD8, to identify the major subsets, we found that there was no significant effect of maternal diet on the proportion of DN, DP, and SP CD4^+^ and SP CD8^+^ thymocytes (data not shown). However the proportion of DP CD4^+^CD8^+^ thymocytes significantly decreased with age (*p* < 0.001) (Figure 2B). Next we further analysed the DN subset populations and found that although they were unaffected by maternal diet, the proportion of DN subset cells were significantly affected by age (Table 2). The proportion of DN1 cells was greatest at 23m (*p* < 0.001) whereas the proportion of DN2 cells decreased with age (*p* < 0.001). In contrast the proportion of DN3 cells was unaffected by age, but the proportion of DN4 cells peaked at 3m of age (*p* < 0.05).

**Table 2:** Effect of age and maternal diet on the proportion of DN subset thymocytes

Age	Diet	% DN1 cells	% DN2 cells	% DN3 cells	% DN4 cells
21d	Control	36 ± 2 A	40 ± 3 E	11 ± 2	12 ± 3 J
	PLP	22 ± 3 C	39 ± 5 G	12 ± 2	26 ± 7
3m	Control	24 ± 2 B	18 ± 1 F	16 ± 3	42 ± 5
	PLP	26 ± 5 D	24 ± 2 H	16 ± 3	34 ± 5
23m	Control	57 ± 5	6 ± 2	11 ± 3	27 ± 0
	PLP	54 ± 8	9 ± 3	12 ± 3	25 ± 5

Thymocytes were stained with appropriate antibodies and were gated for CD4^−^CD8^−^. Subsequently the proportion of DN1 (CD44^+^ CD25^−^), DN2 (CD44^+^ CD25^+^), DN3 (CD44^−^ CD25^+^) and DN4 (CD44^−^ CD25^−^) cells were analysed on the BD FACSCalibur flow cytometer. The proportion of DN1 (*p*<0.001), DN2 (*p*<0.001), and DN4 (*p*<0.05) thymocytes varied with age but were not affected by maternal diet. Following a Duncan's post-hoc analysis the proportion of DN1 cells in control animals was higher at 23m compared to 21d (A where p<0.05) and 3m (B where *p*<0.001). The proportion of DN1 cells in PLP animals was also higher at 23m compared to 21d (C where *p*<0.001) and 3m (D where *p*<0.01). However the proportion of DN2 cells decreased with age (*p*<0.001). In control animals the proportion of DN2 cells was lower at 3m compared to 21d (E where *p*<0.001) and was lower at 23m compared to 3m (F where p<0.05). The proportion of DN2 cells in PLP animals similarly was lower at 3m compared to 21d (G where p<0.05) and was lower at 23m compared to 3m (H where *p*<0.01). The proportion of DN3 cells was unaffected by age, and the proportion of DN4 thymocytes peaked at 3m (*p*<0.05). In control animals the proportion of DN4 cells was higher at 3m compared to 21d (J where *p*<0.01). n = 5-9 per group. Data are represented as mean +/− SEM.

### CD3 expression on developing thymocytes

CD3 expression on thymocytes gradually increases as they progress through this developmental pathway, giving reason to measure the mean intensity of CD3 expression on several cellular populations [16]. We observed that the number of CD3^+^ thymocytes was significantly higher (*p* < 0.05) in the PLP group compared to the control group (Figure 2C). The level of CD3 expression on thymocytes decreased between 3m and 23m of age in both groups (*p* < 0.01) (Figure 2D). However, the rate of decline was markedly different; with CD3 expression levels decreasing by 5-7 fold in control mice, whereas in PLP this was barely 2-fold; thereby highlighting the impact of maternal diet on CD3 expression (Figure 2D). This age-associated decrease in CD3 expression was observed in all major thymocyte subsets (data not shown). However in CD4^+^ thymocytes the age associated decrease in the expression of the CD3 marker (*p* < 0.001) was significantly less substantial in the PLP group (*p* < 0.05) compared to the control group, again suggesting a protection from age-associated changes (Figure 2E).

### Splenic cellularity

**Figure 3:**
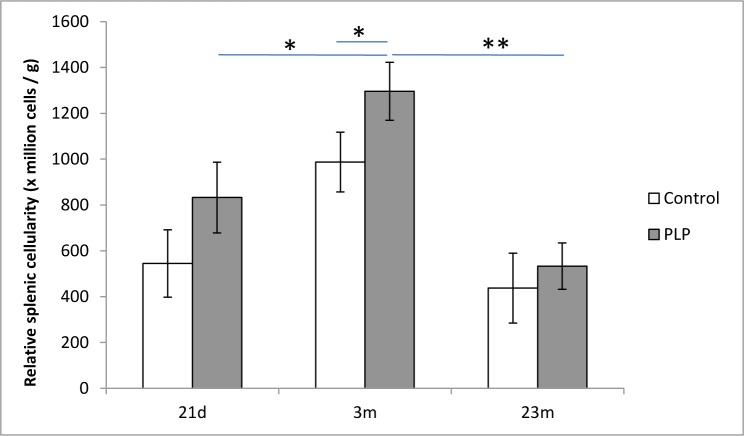
Relative splenic cellularity in aging PLP and control mice In order to detect the effect of age and maternal diet on splenic involution, splenic cellularity was quantified using a Countess Automated Cell counter (Invitrogen, Paisley, UK) and represented in relation to splenic weight. Relative splenic cellularity decreased significantly in old age (*p* < 0.01) and was significantly higher in PLP offspring compared to control animals (*p* < 0.05). *p < 0.05, **p < 0.01. *n* = 5-9 per group. Data are represented as mean +/− SEM.

Given our observation of the effect of age and maternal diet on the thymus, an investigation into the effect on a secondary lymphoid organ was undertaken. Relative splenic cellularity peaked at 3m of age and decreased again by 23m of age which is concurrent with previous findings [23]. It was significantly higher in the PLP group (effect of maternal diet *p* < 0.05) compared to the control group especially at 3m of age (Figure 3). Consistent with previous observations in rodents, splenic weight increased gradually with age but there was no significant effect of maternal diet on this parameter (data not shown) [24]. The total splenocyte T and B cell populations were quantified but were not significantly affected by maternal diet (data not shown).

### Splenic naive and memory CD4^+^ and CD8^+^ T cells

**Figure 4:**
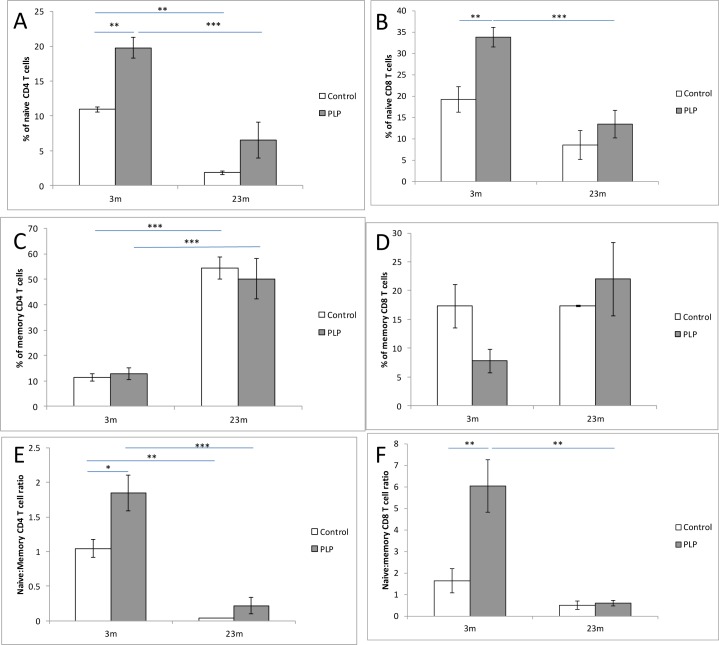
The impact of age and maternal diet on splenic T cells Splenocytes were stained with appropriate antibodies. Cells were gated for either CD4^+^ or CD8^+^ and subsequently the proportion of naive cells (CD62L^+^ CD44^−^) and memory cells (CD62L^−^ CD44^+^) cells were analysed on the BD FACSCalibur flow cytometer. The proportion of naive CD4^+^ cells **A.** and CD8^+^ cells **B.** was significantly affected by maternal diet (*p* < 0.01 and *p* < 0.05 respectively), and was significantly higher in PLP animals compared to controls, especially at 3m of age (**p < 0.01). The proportion of naive CD4^+^ cells and CD8^+^ cells significantly decreased with age (*p* < 0.001). The percentage of memory CD4^+^ cells **C.** significantly increased with age (*p* < 0.001) but the percentage of memory CD8^+^ cells **D.** was unaffected by age. Both parameters were unaffected by maternal diet. The naive:memory CD4^+^ **E.** and CD8^+^ (F) ratios were both affected by maternal diet (*p* < 0.05) especially at 3m of age (*p < 0.05 and **p < 0.01 respectively). This ratio also significantly decreased with age in both cell types (*p* < 0.001 and *p* < 0.01 respectively). *p < 0.05, **p < 0.01, ***p < 0.001. *n* = 5-9 per group. Data are represented as mean +/− SEM.

The observation that PLP animals showed increased relative splenic cellularity compared to control animals may indicate that their spleens demonstrate delayed aging. The splenocyte population was therefore examined on a closer level. Given that the density of splenocytes was very low at 21d, further analysis was only possible at the later time points. The effect of age and maternal diet on naive and memory T cell populations was quantified. The percentage of naive CD4^+^ (*p* < 0.01) and CD8^+^ (*p* < 0.05) T cells was significantly higher in the PLP group compared to the control group especially at 3m of age (Figure 4A and 4B). As expected the percentage of both naive CD4^+^ and CD8^+^ cells decreased significantly with age (*p* < 0.001). The proportion of memory CD4^+^ T cells increased significantly with age (*p* < 0.001) although this population was unaffected by maternal diet (Figure 4C). The proportion of memory CD8^+^ T cells was unaffected by both maternal diet and age (Figure 4D). Overall, these observations meant that the naïve:memory ratio of both CD4^+^ and CD8^+^ splenic T cell were significantly higher in the PLP group (*p* < 0.05) compared to the control group especially at 3m of age (Figure 4E and 4F). As predicted the ratio of naive:memory CD4^+^ and CD8^+^ splenic T cells significantly decreased with age (*p* < 0.001 and *p* < 0.01 respectively). Altogether these results suggest that PLP animals maintain a greater naive T cell population in young and old age compared to control animals.

### Splenic germinal centre (GC) immunohistochemistry

**Figure 5:**
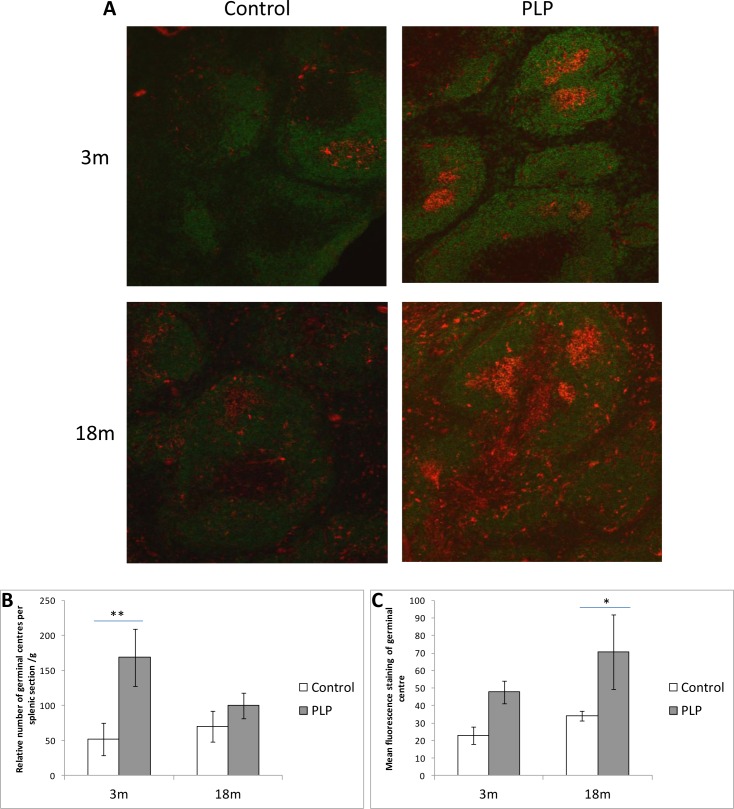
Effect of age and maternal diet on splenic B cell and germinal centre staining **A.** Sections of spleen were fixed in acetone and incubated in PNA biotin conjugate (Vector Laboratories, Peterborough) followed by Streptavidin Alexa Fluor 594 conjugate (Invitrogen, Molecular Probes, Paisley) in order to stain for GCs (red). Double staining was performed where sections were then incubated in a FITC labelled polyclonal anti-B220 antibody (eBioscience). Following staining of 4 sections per animal representing different cross-sections across the spleen, slides were mounted in Vectashield Mounting Medium (Vector laboratories) and visualised on the Zeiss LSM 510 META confocal microscope using Zen 2008 software. **B.** The number of GCs per section was recorded and represented as a proportion of splenic weight. PLP animals had a significantly higher relative density of GCs compared to controls (*p* < 0.05) and this was particularly apparent at 3m of age (**p < 0.01). **C.** The mean staining intensity of GCs was recorded and was found to be significantly higher in PLP animals compared to controls (*p* < 0.01), particularly at 18m (*p < 0.05). However there was no significant effect of age on either parameter. *n* = 6-8 per group. Data are represented as mean +/− SEM.

In order to determine the effect of age and maternal diet on B cell activity, staining of GCs was performed (Figure 5A). PLP animals had a significantly (*p* < 0.05) higher relative number of GCs per section of spleen compared to control animals especially at 3m of age (Figure 5B). The fluorescence intensity of GCs was also significantly (*p* < 0.01) higher in the PLP group compared to the control group especially at 18m of age (Figure 5C). There was however no statistically significant effect of age on these parameters. Taken together these results suggest that PLP animals have greater GC activity compared to controls.

### Splenic gene expression of p16

**Figure 6:**
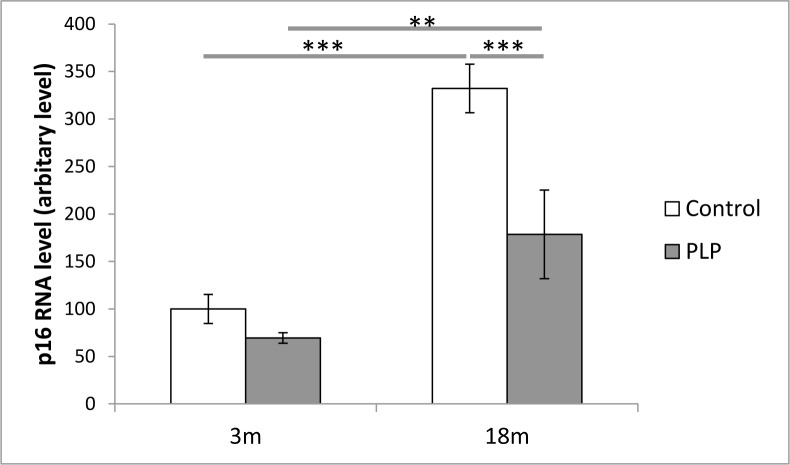
Effect of age and maternal diet on relative gene expression of p16 in the spleen Gene expression levels were measured by quantitative RT-PCR. Values are expressed with mean gene expression in the spleen from 3m control mice being set as 100. Gene expression of p16 was significantly affected by both age (*p* < 0.001) and maternal diet (*p* < 0.001). However PLP animals had a less marked age-associated increase in expression compared to controls. **p < 0.01, ***p < 0.001. *n* = 6-8 per group. Data are represented as mean +/− SEM.

Following our histological findings, splenic aging was investigated on a closer level. RNA levels of p16, a robust biomarker of aging, were quantified by RT-PCR. Gene expression of p16 was significantly affected by both age (*p* < 0.001) and maternal diet (*p* < 0.001). As expected RNA levels of p16 increased with age but were also found to be significantly lower in PLP animals compared to control animals (Figure 6). In addition there was a less marked increased with age in these PLP animals compared to controls (*p* < 0.05).

## DISCUSSION

The thymus is the primary site for development of immunocompetent T cells. In contrast the spleen is a secondary lymph organ that houses both T and B lymphocytes, cells that initiate adaptive immune responses against peripheral antigens. Therefore both organs play an integral part in functional immunity. In rodents and humans the development of the thymus takes place during the late fetal / early postnatal stages of growth, whereas the development of the spleen begins earlier in gestation [23, 25, 26]. Given this, both organs may be vulnerable to early life events. Previous evidence suggests that the thymus and spleen are affected by nutritional insults in early life [6, 13, 27, 28]. We therefore hypothesised that protein restriction during lactation which results in slowed growth and extended longevity may influence thymic and splenic function throughout life.

The present study firstly investigated changes in thymic function in control and PLP animals with increasing age. We observed a decrease in thymic cellularity in old age, indicative of thymic involution. However PLP offspring demonstrated greater thymic cellularity with a greater number of T cells compared to controls. We previously demonstrated that PLP animals had a greater increase in thymic weight between weaning and adulthood compared to control animals [13]. Altogether these parameters suggest a delay in thymic involution in PLP animals. Given the extended longevity observed in these animals, and that thymic involution is a robust biomarker of aging, this suggests a slower rate of thymic aging in the PLP group.

In thymocyte development, pre-TCR expression and signalling occurs at the DN3 stage and progressively increases so that CD3 expression is highest in the most mature thymocytes [16, 17]. The mean intensity of CD3 expression on thymocytes decreased in old age, and this is consistent with previous studies [29]. The age-associated decrease in mean intensity of CD3 expression on SP CD4^+^ thymocytes was significantly less marked in PLP animals compared to controls. Given the crucial role of CD3 in the TCR transduction signalling pathway, a decrease in CD3 expression is likely to negatively impact upon responses to TCR-dependent signals and hence impair thymopoiesis [30]. These results show that PLP animals better maintain CD3 expression on CD4^+^ thymocytes than control animals throughout the life course and could suggest improved TCR signalling and T cell responsiveness in these animals. Here there is evidence that diet in the early postnatal period can modulate the aging trajectory of the immune system and shows protection against the effects of aging.

There were a number of thymic parameters that were only affected by age. The proportion of DN1 thymocytes increased with age whereas the proportion of DN2 thymocytes decreased with age suggesting a developmental block between these stages, again consistent with previous studies [31, 32]. During this stage diversity-joining (D-J) gene rearrangement of the TCR β chain occurs which may be impaired with age [33]. The observed decrease in the proportion of DN4 thymocytes in old age has also been described previously [34]. During this stage mass proliferation and upregulation of CD4 and CD8 receptors occurs, which may also be subsequently impaired with increasing age [17].

Given the observed changes in the thymus, the secondary lymphoid tissue, the spleen was investigated. Splenic development peaks at puberty which is followed by gradual involution throughout life [23]. As reported in previous studies, relative splenic cellularity decreased with age [35]. However relative splenic cellularity was increased at each time point in the PLP group suggesting a slower rate of splenic aging in these animals. Although recent thymic emigrants are fewer in number and diversity with increasing age, homeostatic mechanisms are in place to ensure a constant size of the peripheral B and T cell pool [20]. Therefore PLP animals are likely to require less clonal T cell expansion in order to maintain the same peripheral T cell numbers with increasing age.

In advancing age fewer naive T cells are released from the thymus and so the number of memory T cells increase in the periphery. This results in the loss of TCR diversity in aged individuals and is further exacerbated by homeostatic clonal expansion [36]. Furthermore memory T cells dampen the thymic production of naive cells, thereby perpetuating the proportion of memory T cells in the periphery [37]. This phenomenon is responsible for the increased incidence of neoplasia and infection observed in the elderly. For example the shift in balance from naive to memory T cells is a mechanism that contributes towards the spontaneous incidence of Hodgkin's-like lymphoma in aged SJL/J mice [38]. We observed a significantly higher proportion of naive CD4^+^ and CD8^+^ T cells and a significantly higher ratio of naive:memory CD4^+^ and CD8^+^ T cells in PLP animals. This is likely due to the increased thymic cellularity observed in PLP animals and may suggest that PLP animals would be better protected from a pathogenic challenge and may be less susceptible to cancers. In order to directly determine the effect of maternal diet on T cell mediated immune responses, it would be necessary to infect animals with a viral challenge and measure differences in morbidity and mortality in animals.

The functioning of peripheral immune cells is greatly affected by splenic architecture which was therefore investigated on a histological level. GCs are sites of B cell activation where naive B cells enter and undergo clonal expansion and somatic hypermutation in the dark zone, followed by affinity maturation, T cell-and dendritic cell-dependent selection and class-switching in the light zone [39]. Following this, cells either generate into antibody secreting plasmablasts or form early memory B cells and enter the periphery [40]. Animals were kept in the same conditions with a low level of pathogens. Our results showed an increase in staining and density of GCs in PLP animals compared to control animals which may indicate increased B cell activity in these animals.

p16 is a senescence-promoting tumour suppressor protein that inhibits the cell cycle and has been linked to cancer and aging of nearly all mammalian tissues. It is not only associated with aging but does appear to play a causal role in some tissues [41]. The observed decrease in p16 gene expression in spleens from PLP animals suggests a slower rate of splenic aging in comparison to control animals, and indicates that maternal diet can better protect against age-associated alterations.

The developmental origins of health and disease hypothesis describes the phenomenon whereby the environment experienced in early life can influence the long-term health of the individual. The current findings support the hypothesis that as well as affecting metabolic parameters, early nutrition can impact upon immune function. The observed delay in aging of both the central and peripheral immune systems in animals exposed to reduced protein during the lactation period, may contribute to the increased lifespan observed in these animals. The suckling period may therefore represent a tractable period for intervention to reduce the burden of age-associated diseases.

## MATERIALS AND METHODS

### Mice

All procedures on animals were conducted under the British Animals (Scientific Procedures) Act (1986). C57BL/6 mice were bred at a designated animal unit at University of Cambridge. They were housed at 22°C on a controlled 12h light/dark cycle with access to standard laboratory chow and water. They were mated at 6 weeks of age and females were assumed to be pregnant when a vaginal plug was expelled. Dams were fed *ad libitum* either a control diet (20% protein) or an isocaloric low protein diet (8% protein) during gestation and lactation. These diets were purchased from Arie Blok (Woerden, the Netherlands) and their compositions were described previously [22].

Two days after birth, cross fostering techniques were utilised to establish two groups: The control group (offspring born to and suckled by dams fed a control diet) and the PLP group (offspring of control dams cross-fostered and suckled by dams fed the low protein diet). In order to maximise the effect of maternal diet on differences in offspring postnatal growth control litters were culled to a size of 6 pups, whereas PLP pups were left unculled. Male offspring were fasted overnight and culled using CO_2_ at 21d (weaning), 3m (young adult), 18m (old adult) and 23m (severely old age). Fresh thymic and splenic tissues were immediately removed, weighed and used for flow cytometry analysis. Splenic tissues were snap-frozen in liquid nitrogen and stored at −80°C until use.

### Flow cytometry analysis

Cellular preparation and staining was performed as described previously [42]. Briefly thymi and spleen were teased apart and cell suspensions were collected in PBS. Splenic cells were incubated in red blood cell lysate before cellularity of both suspensions was quantified using a Countess Automated Cell Counter (Invitrogen, Paisley, UK). Cells were stained with FITC (fluorescein isothiocyanate)-, PE (phycoerythrin)- and PerCP-Cy5.5- labelled polyclonal antibodies against the following markers: CD3, CD4 and CD8, and isotype controls (in both thymus and spleen cells); CD44, CD4, CD8 and CD25 (thymus cells); CD3 and B220 (spleen cells); CD44, CD4 and CD62L (spleen cells); and CD44, CD8 and CD62L (spleen cells). All antibodies were purchased from eBioscience (Hertfordshire, UK). Flow cytometry data were collected using a BD FACSCalibur flow cytometer and appropriate gating was performed. Data were expressed as the percentage of gated live lymphocytes which was determined according to forward and side scatter (FSC/SSC).

### Immunofluorescence

Spleens were sectioned (7μm thick), air dried overnight, fixed in acetone and stored at −20°C. Sections were stained as previously described [43]. To detect GCs, sections were incubated in PNA biotin conjugate (Vector Laboratories, Peterborough, UK) appropriately diluted in PNA buffer (10mM HEPES, 0.15M NaCl, 0.1mM Ca^2+^, pH 7.5) for 1h at room temperature. Following three washes in PBS, slides were incubated in Streptavidin Alexa fluor 594 conjugate (Invitrogen, Molecular Probes, Paisley, UK) appropriately diluted in PBS for 1h in the dark at room temperature. Double staining was performed in order to detect B cell regions. Here sections were additionally incubated in rat polyclonal anti-mouse B220 FITC conjugate antibody (eBioscience, Hatfield, UK) diluted appropriately in PBS. Negative controls consisted of primary incubation with 0.5% BSA followed by isotype control antibodies with no staining detected.

Following staining of 4 sections per animal representing different cross-sections across the spleen, slides were mounted in Vectashield Mounting Medium (Vector Laboratories, Ltd. Peterborough, UK) and visualised on the Zeiss LSM 510 META confocal microscope using the software Zen 2008 (Zeiss, Cambridge, UK). 3 images were taken of each section in order to obtain representative staining. The number of GCs per section and the mean staining intensity were quantified (Zen 2008).

### Extraction of total RNA and cDNA synthesis

Total RNA samples were prepared using Trizol reagent (Sigma), then purified using RNeasy Mini Kits (Qiagen) which included a DNase digestion step to eliminate potential contaminating DNA. These were then quantified using a NanoDrop ND-1000 (ThermoScientific). First strand cDNA was reverse-transcribed from 1μg of total RNA using an ImProm-II Reverse Transcription System (Promega) with oligo(dT)_15_ as the primer, according to the manufacturer's protocol.

### Gene expression analysis by RT-PCR (real-time PCR)

Quantitative RT-PCR was carried out using an ABI PRISM 7900 Sequence Detection System (Applied Biosystems) with a SYBR Green PCR Master Mix (Applied Biosystems) and gene-specific primers. Primers were custom-designed and synthesised by Sigma. For p16 the forward primer was CTTTGTGTACCGCTGGGAAC and the reverse was GCCGGATTTAGCTCTGCTCT. For Cyclophilin A the forward primer was CTTGCTGCAGCCATGGTCAA and the reverse was GTCTGCAAACAGCTCGAAGG. cDNA template (3μl; diluted according to the relative expression level of the gene of interest) was used in a 12μl total reaction volume in each well in a 96-well reaction plate. A dissociation curve analysis was also performed to ensure primers did not form primer dimers. The transcripts were amplified in duplicate and standard curves were drawn using serially diluted pooled cDNA samples from each animal. Relative expression levels were calculated against the gene's standard curve with the C_t_ values of each animal, using the housekeeping gene Cyclophilin A as a loading control. Cyclophilin A expression was equal between control and PLP animals at all ages.

### Statistical analysis

A one-way or two-way ANOVA was performed using Statistica (StatSoft Ltd, Milton Keynes, UK) with maternal diet and age as the independent variables as appropriate. A Duncan's post-hoc test was used if appropriate and displayed on the figures and tables. A *p* value of < 0.05 was considered statistically significant.
